# Enferplex bovine TB antibody test and bovine TB diagnosis: letter to the editor

**DOI:** 10.1007/s11259-023-10229-4

**Published:** 2023-10-07

**Authors:** Neil Watt, Alastair Hayton, Keith Cutler, Amanda O’Brien, John Clarke, Gordon D. Harkiss

**Affiliations:** 1 MV Diagnostics Ltd, Roslin Innovation Centre, University of Edinburgh, Easter Bush, Midlothian, EH25 9RG UK; 2 SureFarm Ltd, The Transmission Hall, Rampisham Business Centre, Rampisham Down, Maiden Newton, Dorset, DT2 0HS UK; 3 Enfer Scientific ULC, M7 Business Park, Newhall, Naas, Co., Kildare, W91 FD74 Ireland

**Keywords:** Antibodies, Bovine, Tuberculosis, Diagnosis, Tuberculin, Interferon

## Abstract

Bovine tuberculosis is usually diagnosed using tuberculin skin and interferon gamma tests. However, it is clear these tests miss infected animals due to poor sensitivity. The Enferplex Bovine TB antibody test has been validated by the World Organisation for Animal Health as fit for purpose in diagnosing bovine TB. A recent paper by Madden and colleagues (Veterinary Research Communications published online 17 August 2023) presented data on the future risk of Enferplex test antibody positive animals developing bovine TB. We argue in this communication that this does not make sense. Also, the study design did not include measuring antibodies at the point of censure of the animals and hence the survival analysis performed was meaningless. Most significantly, the study misses the point that skin and interferon gamma tests fail to detect a significant proportion of infected animals identified by the Enferplex test.

## Introduction

Bovine tuberculosis (bTB) remains a major health problem worldwide. Diagnosis of bTB is usually performed using a tuberculin skin test such as the Single Intradermal Comparative Cervical Tuberculin (SICCT) test with the interferon gamma (IFNγ) test used as an adjunct test. It is recognised that these tests suffer from lack of sensitivity and also specificity in the case of the IFNγ test. Recently, antibody tests have been developed which are aimed at increasing the sensitivity of bTB testing. The Enferplex Bovine TB antibody test has been validated by the World Organisation for Animal Health (WOAH) as fit for purpose for diagnosing bTB (within the specificity limits of 98.4% and 99.7% using the high sensitivity and high specificity settings of the test). Thus, by WOAH definition, Enferplex positive animals are deemed to be infected with *Mycobacterium bovis* (*M. bovis*). A recent paper by Madden and colleagues (2023) aimed to assess the association between Enferplex test results and the future risk of bovine tuberculosis in infected herds. In this communication, we comment on the study design, results, and conclusion of this paper.

## Methods

All materials and methods used can be found in the Madden et al. (2023) paper (10.1007/s11259-023-10200-3). Here, we focus on the work performed by Madden and colleagues and comment on the methods used and the authors’ interpretation and discussion of results.

## Results and discussion

The paper by Madden et al. aimed to “…detect additional infected animals that were not picked up by the current standard test regime (i.e., diagnostic testing in parallel)” by identifying “The bTB status of each animal, as determined by the SICCT test, IFNγ or detection of visible lesions at routine slaughter,[…] at the end of a two-year period of follow-up.” The method to assess this was a survival analysis according to the following definition: “Study animals were then followed-up from the enrolment date until the end of follow-up (either date of a positive bTB diagnosis or date of right-censoring (either date of death as a result of routine slaughter, for reasons unrelated to bTB diagnosis or date of end of study (17th February 2022), whichever occurred first).”

Unfortunately, the authors fail to mention that the Enferplex test is validated by the WOAH as fit for purpose for diagnosing bTB. The test performance characteristics have also been peer reviewed and published in Nature Scientific Reports 2023. Enferplex positive animals are, therefore, by definition, infected. That is, the authors (mis)use a test for detecting infection for studying survival to an infection that has a low rate of mortality even if such mortality is caused by reactivity to a highly specific but poorly sensitive test on the negative reactors to previous testing. This is misleading and implies an out of specifications use of a diagnostic tool that undermines the scientific basis for the paper.

The authors also failed to test SICCT test/IFNγ reactors with Enferplex when they were detected over the two-year period. Inevitably, as infection spreads from the Enferplex positive animals to animals which were previously Enferplex negative over the two-year period under investigation they fail to pick this up. According to the WOAH validation, had they done this final test on the SICCT test/IFNγ reactors at least 90–95% of them would be predicted to have been Enferplex positive.

The authors essentially use a positive SICCT test as the determinant of bTB diagnosis. However, since the Enferplex positive animals already had bTB at the start of the study, the authors are really assessing whether any Enferplex positive animals go on to develop evidence of cell mediated immunity, i.e., switch from Th2/T follicular (Th2/Tf) responses to Th1 responses. This also applies to identifying animals with visible lesions (VL) and/or which are *M. bovis* culture positive, since these are effectively manifestations of Th1 T cell responses. The only right and accurate way to evaluate a test is to use a good gold standard. In this case, the authors have used no such standard and have restricted themselves to running a frequency analysis on simple survival that is non-conclusive both because of a small effective sample size lacking statistical resolution power (57 positives versus 427 censored survivors) and an inconceivable inadequate infection standard.

The number of relevant animals in the analysis is very low, and too low to be meaningful (the numbers of VL and *M. bovis* culture positive animals is so low as to be anecdotal). Only 57 animals fulfilled the conditions of a survival analysis, while the vast majority were not examined at a significant endpoint, were not Enferplex-tested or post-mortem pathologically or microbiologically examined at that endpoint and were simply censored. Considering that bTB is a generally subclinical infection that does not cause a lethal disease or production losses requiring culling the animals, it is unlikely that there will be different survival times for infected or non-infected animals.

Chronically infected herds tend to show low numbers of SICCT test reactors, and hence VL/*M. bovis* culture positives. It is known that short interval testing results in the difference in skin thickness between PPDa and PPDb responses decreasing steadily (Coad et al., 2010) and is likely to result in negative SICCT test responses with further short interval testing. Also, chronic antigen presentation is known to lead to immunological regulatory responses which down regulate Th1 cell responses (Wherry 2011; Wherry & Kuachi, 2015; Khan et al. 2017). Thus, using Th1 measures to detect infected animals in chronic herds will suffer lower sensitivity due to highly immunoregulated T cell responses. The use of both bovine and avian tuberculin in the SICCT and IFNγ tests is also known to result in increased specificity but reduced sensitivity of the tests. Hence, the number of animals shown to develop a positive skin test should predictably be an underestimate due to this. These observations probably explain the low number of SICCT test positive animals detected and the negligible numbers of VL/*M. bovis* positive animals in this study. None of these points appear to have been considered at the study conception and are not addressed by the authors in the discussion of results.

While the question of whether Enferplex antibody positive animals go on to develop Th1 responses is interesting, it is not the one posed by the authors, and this results in misleading readers as to the value of antibody testing. There is no *a priori* reason why antibody (Th2/Tf) responses should go on to switch to Th1 responses – some will, some won’t, depending on the host/infection dynamics. The authors recognise this but go on to state that since there is no ‘perfect diagnostic test, infection status was inferred using existing tests accepting their limitations’. No mention is made of the fact that the Enferplex is a WOAH validated test for diagnosing bTB. It is not clear why the authors think that they can make definitive judgements about ‘future risk of bTB in Enferplex positive animals’ using Th1 responses if they accept that they cannot ‘assume that antibody tests will always test SICCT test or IFNγ positive’. The authors appear to accept that they can’t answer their primary question using existing tests but go on to do just that.

The authors state that the Enferplex test detected 35.3% and 31.1% of SICCT test negative and IFNγ test negative animals as positive for antibodies using the high sensitivity and high specificity settings of the test respectively at the start of the study. These animals, by WOAH definition, have bTB and were missed by both SICCT and IFNγ tests. No mention or discussion of this is made in the paper. This is the epistemologic nub of the issue since the authors fail to apply the scientific method reasoning. It is recognised that the CMI tests miss truly infected animals and that new diagnostic tests are required to fill this gap, yet they use these techniques to infer the infection status as a reference for the Enferplex test utility. The Enferplex test has been shown to detect infected animals missed by SICCT testing and IFNγ (O’Brien et al. 2023). In our experience, chronic bTB herds often have 20–30% (sometimes more) Enferplex positives, so 35% positivity is not unusual. For example, DEFRA study SE3263 showed that 10% of 1012 SICCT test negative, IFNγ negative animals in infected herds responded positively in the version of the Enferplex which was used in 2012. We have improved the sensitivity of the Enferplex test with the WOAH-validated version and testing the same samples shows that 23% were positive. We will be submitting this work for publication shortly.

The authors suggest that some animals may have developed an antibody response resulting from multiple tuberculin injections, i.e., that some of the antibody responses are false positives. There is no evidence that injection of tuberculin on its own in the absence of *M. bovis* infection induces antibodies and there are several publications in the literature that report the opposite, i.e., that tuberculin injection does not induce *de novo* antibody responses in bTB free animals. Thom et al. (2004) investigated repeat SICCT testing in uninfected cattle on weeks 0, 8, 16, 24, and 32, taking blood for antibody testing at four weekly intervals and 1 week after each SICCT test (i.e., in the period where it would be expected that antibodies would be boosted by the anamnestic response to the injected tuberculin). No antibodies were detected after repeat SICCT tests but were found approximately two weeks after *M. bovis* challenge at week 40. This publication was not quoted in the paper.

All cattle in the UK and Ireland undergo SICCT testing at intervals ranging from 6 months – 4 years over their lifetime. If tuberculin injection induced antibody responses, then it would be expected that a substantial proportion of the national UK and Irish herds would show antibodies if tested. This is not the case. Enferplex specificity was found to be 98.4% and 99.7% at the high sensitivity and high specificity settings of the test. Boosting with tuberculin did not affect the specificity. Differences in specificity between UK, EU and USA herds were minor.

Induction of immune responses requires antigen being presented to competent T and B cells along with a ‘danger’ signal. The latter is recognised by antigen presenting cells through pattern associated molecular receptors (PAMPs) such as toll-like receptors (TLR) and mannose receptors (Kawai and Akira 2011). Without this second signal T cells (and hence B cells which generate antibodies) are not activated. Tuberculin is primarily a protein antigen which is devoid of pattern recognition molecules capable of engaging TLR and the like. Hence, injection of PPDb does not induce T helper cell responses in bTB free animals and hence no memory cells which are required for anamnestic antibody responses. The authors did not quote the existing literature when they suggested multiple tuberculin testing induced antibodies.

Supplementary Fig. 1 (and Table 2) show that only a small fraction of highly antibody positive animals (including the top quartile) is picked up by the official tests. This is not commented on by the authors. As mentioned above, neither poor specificity nor tuberculin boosting provides an adequate explanation for such results, and it is more likely that the CMI tests are missing truly infected animals. The difficulty of eradicating bTB is that only a few animals are picked up with the most sensitive official techniques which, in Ireland, are the SICCT test and the IFNγ release assay, both CMI tests. This is the standard worldwide WOAH supported diagnostic strategy even though the intradermal test might be replaced by the single caudal fold or cervical inoculation in some countries or regions. Even though adding the IFNγ test might slightly improve sensitivity, the IFNγ test still is a cellular comparative test subject to avian TB interference and potential masking of bTB infected individuals. DAERA estimate that ‘approximately 20% of TB-infected cattle can be missed by one round of skin testing using standard interpretation (DAERA website).

In summary, we think this paper is poorly conceived, misleading, and grossly fails to address the main issue which is detection of residual infection missed by the SICCT and IFNγ tests. This missed infection leaves up to one third of the herd as a reservoir of infection in the herds undergoing CMI tests, that could have been picked up by the Enferplex test. This underlines the complementary role the Enferplex could play in the TB eradication programs. We think it is urgent to add an antibody detection test such the Enferplex test to current TB eradication programs to stop wasting huge amounts of public resources by not increasing the sensitivity of current official tests.

## Data Availability

Not applicable.
